# Bioinformatics Analysis and Experimental Verification of Exercise for Aging Mice in Different Brain Regions Based on Transcriptome Sequencing

**DOI:** 10.3390/life13101988

**Published:** 2023-09-29

**Authors:** Yu Jin, Changling Wei, Xiaohan Huang, Deman Zhang, Li Zhang, Xue Li

**Affiliations:** 1 School of Sport Medicine and Health, Chengdu Sport University, Chengdu 610041, China; jinyu@cdsu.edu.cn (Y.J.); weicl96@126.com (C.W.); m15319319828@163.com (X.H.); zdedem@126.com (D.Z.); 2 Joint International Research Laboratory of CNS Regeneration, Guangdong-Hong Kong-Macau Institute of CNS Regeneration, Jinan University, Guangzhou 510632, China; zhangli@jnu.edu.cn

**Keywords:** exercise, aging, RNA-seq, hippocampus, PFC

## Abstract

Purpose: Physical exercise mitigates the effects of aging and cognitive decline. However, the precise neurobiological mechanisms underlying this phenomenon remain unclear. The primary aim of this study was to investigate the protective effect of exercise on age-related memory deficits in the prefrontal cortex (PFC) and hippocampus using bioinformatic analysis and biochemical verification. Methods: Young and aging mice were subjected to natural feeding or treadmill exercise (12 m/min, 8 weeks). Cognitive function was accessed using the Barnes maze and novel object recognition. Bioinformatic analysis was performed to identify co-expressed genes in different groups and brain regions. The selected genes and pathways were validated using RT-qPCR. Results: Regular exercise significantly ameliorated age-related cognitive deficits. Four up-regulated targets (Ifi27l2a, Irf7, Oas1b, Ifit1) and one down-regulation (Septin2) were reversed by exercise, demonstrating the underlying mechanisms of cognitive functions induced by aging with exercise in the hippocampus and PFC. The Gene Ontology terms and Kyoto Encyclopedia of Genes and Genomes pathway enrichment analyses indicated that the NOD-like receptor signaling pathway was inhibited in the neuroinflammation effects of exercise in aging mice in both brain regions. Conclusion: Exercise enhances age-related learning and memory deficits. This beneficial effect may be attributed to the changes in five up/down-regulated genes and the NOD-like receptor signaling pathway in both the hippocampus and PFC. These findings establish the modulation of neuroinflammation as a pivotal molecular mechanism supporting exercise intervention in the brain aging process.

## 1. Introduction

Aging is a primary risk factor for chronic human disease. According to the United Nations, between 2019 and 2050, the global population aged 65 and above is anticipated to exceed 1.5 billion individuals, constituting 16% of the world’s population [1,2]. Aging causes a multitude of severe systemic complications that significantly affect various organs, with a notable impact on the brain. Brain aging can lead to cognitive impairment and increased vulnerability to neurodegenerative diseases (NDDs) such as Alzheimer’s disease (AD). These alterations primarily occur in the hippocampus and prefrontal cortex (PFC) due to aging, resulting in cognitive deficits and memory dysfunction in humans [3,4]. Nevertheless, the intricate neurobiological mechanisms underlying cognitive dysfunction in relation to aging remain unclear. Presently, available treatments fail to halt or decelerate the detrimental progression of AD associated with aging, primarily because of the unclear molecular mechanisms involved in AD pathogenesis. An in-depth understanding of these mechanisms could offer invaluable strategies to mitigate the aging process and prevent the onset of NDDs.

Several beneficial lifestyles have been substantiated to rejuvenate cells and organs, delay the onset of aging and NDDs, and extend healthy lifespans. Among these, vigorous physical exercise has emerged as one of the most efficacious and healthy intervention measures [2]. Exercise exerts a positive influence on brain structure and function, particularly in older adults, facilitating the restoration of cognitive functions, including tasks reliant on the hippocampus and the maintenance of overall brain health [5,6]. The observed inverse relationship between physical exercise and cognitive deficits during aging implies that exercise-induced molecular biological changes during the aging process may constitute a significant event linked to the restoration of cognitive function. Numerous studies have investigated the mechanisms involving reduced oxidative damage molecules, diminished inflammation, intracellular signal transduction, alterations in gene expression, enhanced neurogenesis, and synaptic plasticity, all of which are relevant to the response to age-related changes in cognitive function [7,8,9]. Despite previous investigations in the field of neurobiology pertaining to aging and exercise, a comprehensive network of gene expression, particularly through transcriptome and high-throughput sequencing, especially within two distinct brain regions, has yet to be established. Consequently, we integrated information gleaned from transcriptomes across multiple brain tissues to probe the effects of aging and its regulation through treadmill exercise using bioinformatics methodologies in the Mus musculus mammalian model. Subsequently, behavioral tests and biochemical experiments were performed to validate alterations in phenotypes and gene expression levels. For instance, real-time quantitative polymerase chain reaction (RT-qPCR) has been used. The aim of these research efforts is to assess the potential advantages of treadmill exercise in mitigating the effects of aging in mice.

## 2. Results

### 2.1. Aerobic Exercise Ameliorated the Deficits of Cognitive Function in Barnes Maze and Novel Object Recognition (NOR) Test

**Figure 1:**
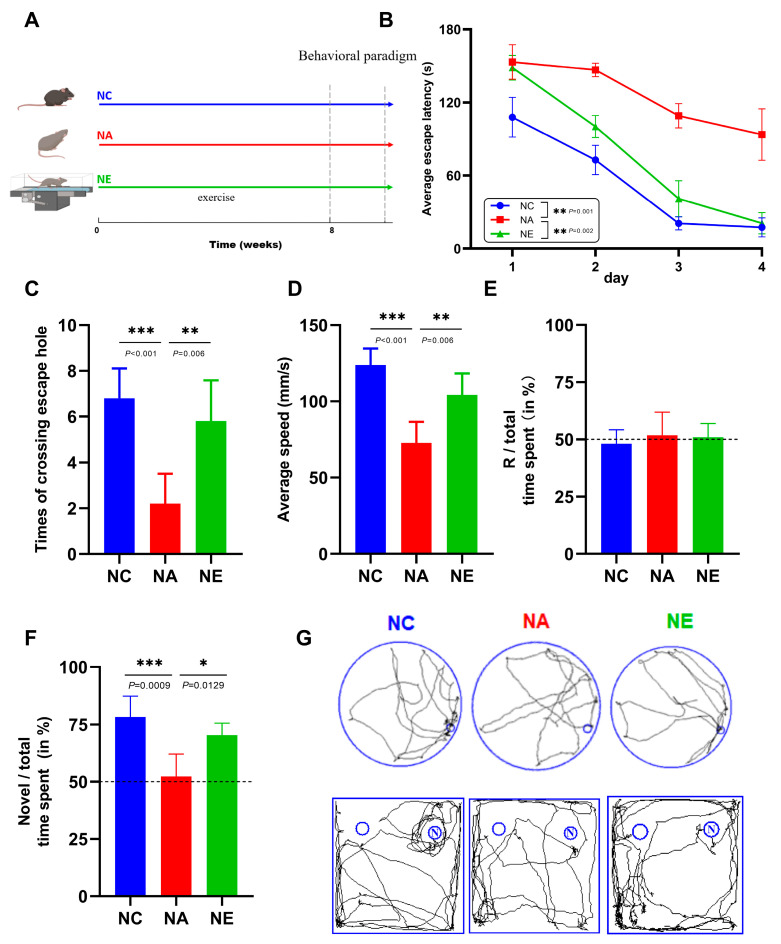
Exercise ameliorated deficits of cognitive functions for aging mice in behavioral test. NC group, young mice; NA, aging mice; NE, aging mice while undergoing eight weeks of exercise intervention. (**A**) Timeline for experimental procedures. (**B**) Escape latency on the first–fourth day of each group during the training session in Barnes Maze. (**C**,**D**) Times of crossing the escaped hole (**C**) and average speed of each group on the 5th day from the Barnes maze (**D**). (**E**,**F**) Percentage of time spent exploring objects on the right side during the learning session (**E**) and preference of mice for the novel object in the testing session as calculated by the ratio of novel object approaching time against total approaching time (**F**). (**G**) Representative trajectories of Barnes maze (top) and NOR (bottom, N means novel object). Data were shown by M ± SD, * *p* < 0.05, ** *p* < 0.01, *** *p* < 0.001, respectively. *n* = 5 animals per group.

To evaluate whether treadmill exercise confers cognitive benefits, we subjected aging C57BL/6 mice to the behavioral test (Figure 1A), which is an established paradigm designed to assess age-related impairments in spatial cognition and working memory. Notably, aging mice exhibited a lack of improvement in their learning performance over the course of the 4-day training period (Figure 1B, *p* < 0.01). In stark contrast, aging mice that underwent exercise treatment demonstrated escape latencies in the Barnes maze comparable to those of control mice on days 1–4 (Figure 1B, *p* > 0.05). During the testing phase, significant differences were evident between the various groups of mice (Figure 1C,D, *p* < 0.05). The mice in the NA group exhibited notably inferior performance in terms of the number of times they crossed the escape hole compared to the NC group (Figure 1C, *p* < 0.001). Conversely, treadmill training significantly increased the number of times mice crossed the escape hole on the fifth day (Figure 1C, *p* < 0.01). Moreover, the average speed of mice in the NA group was significantly decreased (Figure 1D, *p* < 0.001), whereas it markedly increased in the NE group (Figure 1D, *p* < 0.01). We also investigated memory functions by performing NOR tasks on aging mice receiving treadmill exercise. It was observed that neither group showed any alteration in location preference during the learning phase (Figure 1E, *p* > 0.05). However, the aging mice demonstrated a decrease in the time spent exploring the novel object, which was subsequently restored following treadmill exercise during the testing phase (Figure 1F, *p* < 0.05). These data showed that the impaired working memory in aging mice can be rescued by physical exercise. The trajectory map further confirmed that exercise-trained mice crossed the target hole and had a preference for novel objects more frequently than their aged counterparts (Figure 1G). Collectively, these data indicate that exercise intervention substantially ameliorates cognitive declines in aging mice.

### 2.2. Conversion of Aging Relevant Gene Expression across Brain Area by Exercise

**Figure 2:**
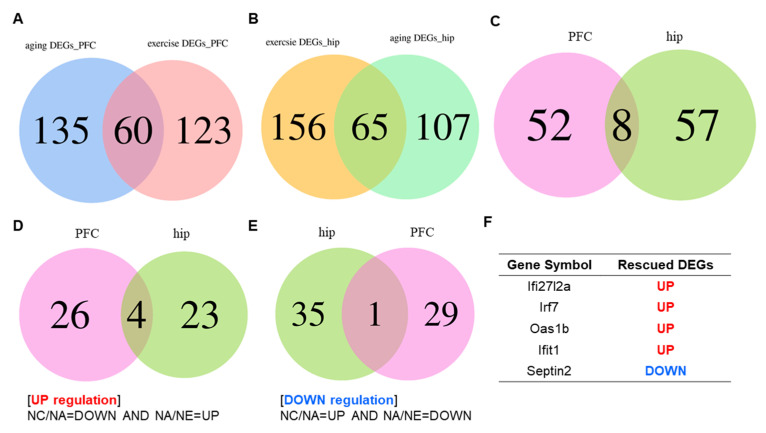
(**A**,**B**) Venn diagrams showing the numbers of “aging DEGs” and “exercise DEGs” in the PFC (**A**) and hippocampus (**B**). (**C**) Venn diagrams showing overlapping DEGs in two brain regions. (**D**,**E**) The overlapping area showing the numbers of up-regulated rescued DEGs (**D**) and down-regulated rescued DEGs (**E**) in the PFC and hippocampus. (**F**) The detailed information of the 5 rescued DEGs in the PFC and hippocampus. *n* = 3 animals per group.

To elucidate the neuromolecular events associated with aging and exercise, we identified and designated the differentially expressed genes (DEGs) between NA and NC mice and between NE and NA mice as “aging DEGs” and “exercise DEGs” in distinct brain regions, respectively (Figure 2A,B). The PFC exhibited 195 aging DEGs and 183 exercise DEGs. The results of the correlational analysis revealed that 60 target genes associated with aging were also linked to exercise, with detailed data on the 60 genes highlighted in Table S1 and Figure 2A. In the hippocampus, there were 172 genes identified as aging DEGs and 221 genes as exercise DEGs, with 65 genes overlapping between exercise and aging (Figure 2B, Table S2). Based on this, we conducted a visual analysis of co-expressed genes in both the PFC and hippocampus, revealing that the abundance of eight factors (Figure 2C), Septin2, Ifit1 (tetratricopeptide repeats 1), Irf7 (interferon regulatory factor 7), Ifi27l2a (interferon alpha-inducible protein 27 like 2A), Oas1b (2′-5′ oligoadenylate synthetase 1 B), Rn7sk (RNA component of 7SK nuclear ribonucleoprotein), Zfp62 (Zfp62 zinc finger protein), and Fsd1l (fibronectin type III and SPRY do-main containing 1 like), exhibited alterations.

Subsequently, a comparative analysis of DEGs was performed to further explore aging DEGs that were partially rescued by exercise, referred to as “rescued DEGs”, encompassing both upregulated and downregulated genes. The criteria for defining these genes are illustrated in Figure 2D,E. As depicted in Figure 2D, there were 30 upregulated genes in the PFC and 27 upregulated genes in the hippocampus, resulting in the identification of four co-expressed genes between the two brain regions (Figure 2D). Conversely, 30 downregulated genes were identified in the PFC and 36 were identified in the hippocampus, yielding a single overlapping gene between the two regions (Figure 2E). Remarkably, Venn diagrams revealed that the five genes exerted the most pronounced influence on both aging and exercise, as evidenced by the shared rescued DEGs in the PFC and hippocampus. Detailed information regarding these five genes is shown in Figure 2F.

### 2.3. GO Terms and KEGG Pathway Enrichment Analyses

To illustrate the biological characteristics of bioinformatics targets influenced by exercise in an aging model, we conducted Gene Ontology (GO) terms and Kyoto Encyclopedia of Genes and Genomes (KEGG) pathway enrichment analyses using Omic Studio online tools. The results of the initial analysis of the GO information are presented in Tables S3 and S4. The data reveal the most significantly enriched terms in the Biological Processes (BP), Cellular Components (CC), and Molecular Functions (MF) categories, indicating that youth and exercise may enhance cognitive function in aging mice in both PFC (Figure S1) and hippocampus (Figure S2) by modulating processes related to the immune system and the innate immune response.

**Figure 3:**
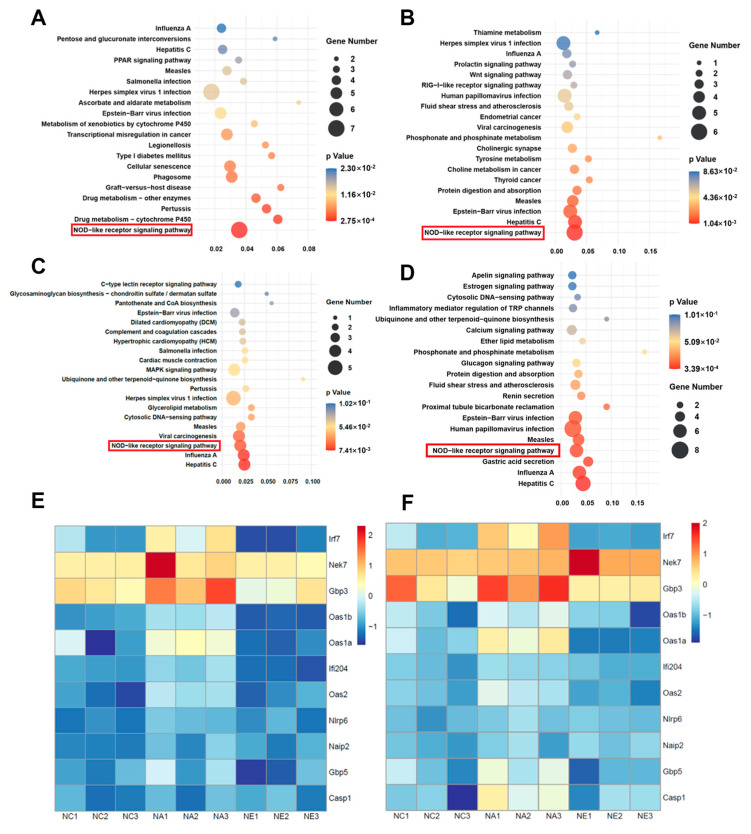
(**A**–**D**) KEGG enrichment of differentially expressed genes identified in NC vs. NA (left) and NA vs. NE (right) and in the PFC (**A**,**B**) and the hippocampus (**C**,**D**). (**E**,**F**) Heatmap displaying up- and down-regulated genes among different groups in the PFC (**E**) and the hippocampus (**F**). *n* = 3 animals per group.

KEGG enrichment analysis, a common approach for identifying signaling pathways affected by treadmill exercise-induced cognitive rehabilitation during aging, was performed. Detailed pathway enrichment information pertaining to exercise intervention is outlined in Tables S3 and S4. The top 20 enriched pathways are shown in Figure 3A–D. The NOD-like receptor (NLR) signaling pathway has emerged as a highly represented pathway in both the PFC and hippocampus. Our attention was specifically directed towards the NLR pathway, and we have depicted a heatmap illustrating differences between various groups in the PFC (Figure 3E) and hippocampus (Figure 3F). The observed levels were corroborated by high gene expression values for genes with age-specific expression and comparatively lower expression in exercise-treated aging mice, both in the PFC and hippocampus (e.g., Irf7 and Oas1b).

### 2.4. Identification of Genes Expression by RT-qPCR

**Figure 4:**
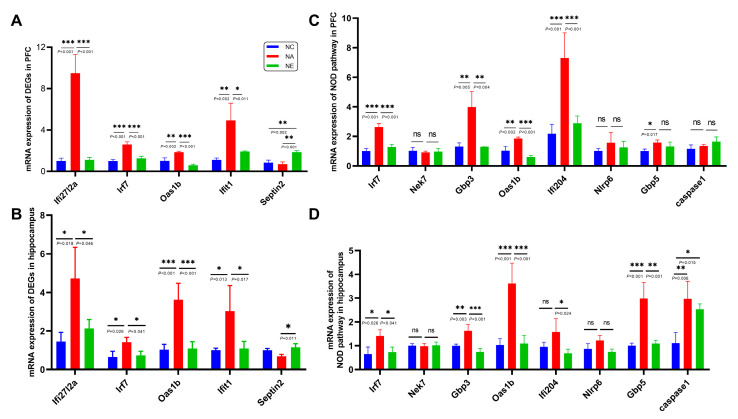
The relative mRNA expression by RT-qPCR. (**A**,**B**) 5 rescued DEGs in the PFC (**A**) and hippocampus (**B**). (**C**,**D**) 8 NOD-like pathway-related genes in the PFC (**C**) and hippocampus (**D**). *n* = 3–4 animals per group. Data were shown by M ± SD, ns no significant difference, * *p* < 0.05, ** *p* < 0.01, *** *p* < 0.001, respectively.

We further validated the expression levels of rescued DEGs identified using sequencing technology. The mRNA expression levels of these five genes (Ifi27l2a, Irf7, Oas1b, Ifit1, and Septin2), confirmed by RT-qPCR, were in agreement with the sequencing results. Compared to the NC group, the expression of Ifi27l2a, Irf7, Oas1b, and Ifit1 in the PFC of aging mice was significantly increased (Figure 4A, *p* < 0.001, *p* < 0.001, *p* < 0.01, *p* < 0.01, respectively). Conversely, eight weeks of exercise treatment significantly attenuated the mRNA expression of these genes induced by aging (Figure 4A, *p* < 0.001, *p* < 0.001, *p* < 0.001, *p* < 0.05). The expression of Septin2 in the NE group was substantially improved by exercise compared to NA groups (Figure 4A, *p* < 0.01), but there was no significant difference between the NC and NA groups (Figure 4A, *p* > 0.05).

In the hippocampus, compared to the control group, the mRNA expression of the rescued DEGs (Ifi27l2a, Irf7, Oas1b, and Ifit1) was significantly increased in aging mice (Figure 4B, *p* < 0.05, *p* < 0.05, *p* < 0.001, *p* < 0.05, respectively), but exercise training reversed these changes caused by aging and notably suppressed the expression of these genes during the aging process (Figure 4B, *p* < 0.05, *p* < 0.05, *p* < 0.001, *p* < 0.05). Septin2 was also elevated following aging mice with exercise intervention (Figure 4B, *p* < 0.05), and no significant difference was observed between the NC and NA groups (Figure 4B, *p* > 0.05). All the RT-qPCR results were consistent with the sequencing results.

KEGG enrichment analysis indicated that the NLR signaling pathway was upregulated by the aging process, and exercise reversed these changes (Figure 3E,F and Figure 4C,D). The mRNA levels of NLR signaling pathway genes (Irf7, Nek7, Gbp3, Oas1b, Ifi204, Nlrp6, Gbp5, caspase1) were also confirmed by RT-qPCR in control, aging, and aging + exercise mice in the PFC and hippocampus. The results indicated that aging induced significant upregulation of mRNA levels of Irf7, Gbp3, Oas1b in both brain regions, these changes were statistically significant and exercise training greatly reverses these changes (Figure 4C,D, *p* < 0.05). Ifi204, which was significantly downregulated by aging with exercise in both regions, was markedly increased by aging in the PFC but showed no statistical differences in the hippocampus. Gbp5 was markedly increased by brain aging (Figure 4C,D, *p* < 0.05), with a decrease observed in the hippocampus (Figure 4D, *p* < 0.01), but no statistical significance was found in the PFC (Figure 4C, *p* > 0.05). Caspase1, in the PFC, showed no significant differences between the groups (Figure 4C, *p* > 0.05). In the hippocampus, caspase1 was upregulated in aging mice compared to the control group, but no statistical significance was found in the aging group compared to the exercise group (Figure 4D, *p* > 0.05). Additionally, the RT-qPCR results for the expression of Nek7 and Nlrp6 were not significantly different among the three groups (Figure 4C,D, *p* > 0.05).

## 3. Discussion

This project was undertaken to assess the impact of exercise on aging mice at the molecular level, using transcriptome sequencing techniques. Our goal was to elucidate the effects of age-related changes in gene expression and transcriptional regulation to collectively provide insights into the mechanisms underlying aging. Furthermore, we aimed to provide strong evidence of the beneficial effects of exercise during the aging process, shedding light on the neural mechanisms that drive both age-related brain dysfunction and brain rejuvenation, ultimately rescuing many age-related phenotypic changes. Additionally, our results identified age-associated changes in different brain regions, including the PFC and the hippocampus. To the best of our knowledge, this is the first study to integrate systematic bioinformatics and conjoint analyses in different brain regions to elucidate the beneficial mechanisms of exercise in the central nervous system.

A substantial body of research has demonstrated that aging and age-related diseases can lead to neurodegenerative changes, including learning and memory deficits in both humans and animal models [10,11,12]. Previous studies have shown that exercise training, including treadmill running, swimming, and voluntary wheel running, can reduce neural inflammation and oxidative stress and improve cognitive deficits in rats and mice [13,14,15,16]. In an aging rat model induced by D-galactose, 6–8 weeks of moderate-intensity exercise training improved spatial learning and memory [15,17]. Voluntary exercise mitigated cognitive deficits and suppressed neural degeneration in the hippocampus of senescence-accelerated SAMP8 mice [18]. We investigated the effects of treadmill exercise on age-related cognitive impairment and reported the underlying neural mechanisms. To assess the ability of mice to learn locations and perform memory tasks, we used the Barnes circular maze and NOR test, which allows assessment through average escape latency, measurements of times crossing the escape hole and preference of novel object. Our results showed impaired hippocampus-dependent and working memory recovered after 8-week exercise training. Therefore, exercise interventions appear to protect neurocognition and alleviate age-related learning and memory dysfunctions.

Numerous researchers have attempted to identify transcriptomic changes during the aging process in various tissues, including humans, monkeys, drosophila, rats, and mice [19,20,21,22,23], using sequencing technology. This idea is supported by numerous studies indicating that aging is closely associated with increased inflammatory levels, rendering individuals more susceptible to various chronic diseases [23,24,25]. Similarly, our analysis revealed an increased number of BP related to the immune system, including immune system processes, innate immune responses, and inflammatory responses, enriched in aging brain tissue, whether or not these changes were reversed by exercise. Recent studies have highlighted the significance of the hippocampus and PFC in learning and memory [26]. Although previous studies have explored the impact of exercise on cognitive function in AD from the perspective of two brain regions [27], our research represents the first integration of transcriptome sequencing technology to observe common characteristics at the genetic level. To gain a better understanding of the region-specific effects of aging with exercise and to identify crucial biomarkers of brain aging with exercise, we analyzed transcriptional and co-expression changes in these brain regions and found that some factors (Ifi27l2a, Irf7, Oas1b, Ifit1, and Septin2) were dysregulated during aging and rescued by treadmill training, contributing to the restoration of these factors in various tissues.

Increased Ifi27l2a expression was observed across the different brain regions examined. This gene encodes the interferon alpha-inducible protein 27-like protein 2a. A recent study on aged mouse brains using single-cell RNA sequencing (sc-RNA seq) reported significant upregulation of Ifi27l2a, particularly in microglia [28]. In AD models, there is a significant increase in the expression of a panel of genes, including Ifi27l2a, Irf7, and Oas1, all of which are vital elements of the neuroinflammatory state of AD and contribute significantly to the neuropathogenic process [29]. We also observed a uniform increase in Irf7 and Oas1b expression in both brain regions during aging. Irf7 belongs to the interferon regulatory transcription factor family and is recognized as a master regulator of type I IFN signaling, which plays a pivotal role in innate and immune responses [30]. The expression of Irf7 may affect brain immune efficiency and is highly correlated with clinical dementia in patients with AD [31]. Research on head and neck cancer patients supports the idea that exercise may decrease inflammation, potentially linked to the downregulation of Irf7 [32]. This study showed increased Irf7 during aging process and down-regulated expression of Irf7 by exercise, as consistent with those mice with caloric restriction [33]. The Oas1b gene is known to be upregulated in virus-caused lethal encephalitis as part of the inflammatory and immune responses in the mouse brain. It has been selected as a potential biomarker for the diagnosis and targeted therapy of virus-induced neurodegeneration [34]. These findings, combined with our own, indicate a clear association between aging and increased expression of Ifi27l2a, Irf7, and Oas1b in brain regions. This raises the possibility that all of these may be a target of interest for exercise training to prevent or treat age-related dementia symptoms.

The product of Ifit1 is an inflammatory protein. We observed upregulation of Ifit1 expression in the aging brain, which was subsequently suppressed by exercise training. Recent data have shown that the levels of innate immune markers, including Ifit1, were significantly increased in virus-infected brains on the 12th day [35]. Another study demonstrated elevated expression of interferon type I mRNA signatures (Irf7 and Ifit1) in older brains compared to younger brains [36]. Furthermore, the expression of this gene is higher in obese patients with lower sports performance, indicating its role in obesity-related inflammation [37]. Our data confirmed that the anti-inflammatory benefits of exercise may be associated with changes in the expression of this immunomodulatory protein. Interestingly, among the aging-induced rescued downregulated genes, only one, Septin2, encodes highly conserved GTP-binding cytoskeletal proteins [38]. It has an extensive range of functions, including cytokinesis, cell division, and exocytosis [39] and is reported to restore cytokinesis in neural progenitor cells in the brain, suggesting a direct mechanism for resisting neural cell death [40]. Combining our research, Septin2 may activate cell proliferation, leading to improvements in learning and memory abilities through exercise during the aging process. The effects of Septin2 on aging and exercise require further investigation. In summary, these findings suggest that regular treadmill exercise may reprogram the regulatory signals associated with aging-related neural inflammation and proliferation, which could be interrelated with exercise during the aging process.

To provide a detailed understanding of the biological characteristics, we conducted enrichment analyses, including GO and KEGG, to elucidate the involved targets. GO enrichment terms indicated that exercise may ameliorate age-induced cognitive deficits through its anti-immune or anti-neuroinflammatory effects. KEGG pathway analysis revealed that regular training may exert beneficial effects on memory impairment, primarily by mediating neural inflammation through the NLR signaling pathway, which regulates the initial innate immune response to cellular injury and stress, playing roles in inflammation-associated tumorigenesis, angiogenesis, cancer cell stemness, and chemoresistance [41,42]. Neuroinflammation is a pathological hallmark of aging and related NDDs [43]. The connection between immune function and the nervous system is increasingly recognized as complex and interrelated [44]. As the principal immune cells within the brain, microglia modulate both the structural and functional aspects of synaptic plasticity [45], neurogenesis [46], protein aggregation [47], and other molecular mechanisms in conjunction with aging and NDD hallmarks to accelerate the disease process. A healthy diet could alleviate chronic inflammation and thus prevent the procession of inflammatory aging [48]. Physical exercise is also shown to be beneficial for primate and rodent health by mitigating age-induced inflammation and cognitive decline. These cognitive benefits are closely associated with increased neuroplasticity and reduced inflammation [49].

Studies have reported that exercise can prevent microglial priming in aged rats and mice. For example, microglia from runner rats exhibited lower expression of certain cytokine mRNA following LPS-induced microglial inflammation in vivo [50]. Running also shifts the microglial phenotype towards neuroprotection in aged mice [51,52]. Recent research suggests that the NLR pathway is a highly dynamic process that can be potentiated by microglial activity in aging mice [53]. However, little is known about the NLR pathway and the mechanisms mediating the effects of exercise training on the central nervous system. To validate the enrichment results further, we examined the main genes involved in the NLR pathway. Our results align with those of RNA-seq analysis, showing that exercise reduces the levels of Irf7, Gbp3, and Oas1b in both brain regions. The level of other proteins (Ifi204, Gbp5, Nek7, Nlrp6, caspase1) exhibits regional specificity, which may be related to the fact that our detection is at the transcription level, and further protein expression and modifications following translation are necessary to exert their effects.

It is important to note that this study has limitations that require further investigation. We used RT-qPCR to verify the mRNA levels of the brain samples in different groups, and western blotting was not employed to measure the relative levels of proteins. Furthermore, young mice with exercise should be taken into consideration to enhance the comprehensiveness of the research. Therefore, the target genes should be further validated in future studies.

## 4. Materials & Methods

### 4.1. Experimental Design and Exercise Protocol

C57BL/6 mice (male, aged 5 weeks and 12 months) were obtained from Chengdu Dossy Experimental Animal Technology Co., Ltd. (Chengdu, China). Mice were housed in mouse cages with a 12-h light and 12-h dark cycle under specific pathogen-free (SPF) conditions. After a one-week acclimatization period, the mice were randomly divided into the following groups, 15 in each group: the natural control group (NC, 5 weeks old), the natural aging group (NA, 12 months old), and the natural aging group with aerobic exercise (NE, 12 months old). The experimental timeline is shown in Figure 1A. The exercise protocol was carried out as previously described [54,55], using a 6-channel treadmill apparatus (SANS Instruments, Nanjing, China) at a speed of 12 m/min for 1 h daily over an 8-week period (5 to 12 m/min, 30 to 60 min/day to adapt the apparatus and avoid exercise-induced stress). After exercise, a portion of the mice was subjected to behavioral experiments, while another portion was utilized for sequencing and mRNA detection. All the animal experiments were approved by the Animal Ethics Committee of Chengdu Sport University (Batch No: 2022-30).

### 4.2. Behavioral Test

#### 4.2.1. Barnes Maze

The Barnes maze consists of a white platform with a diameter of 0.91 m, positioned 0.9 m above the ground. There were 18 evenly spaced escape holes, each with a diameter of 0.05 m. An opaque white escape box (SA208M, SANS Instruments, Nanjing, China) was placed beneath one of these holes. Spatial cues with distinctive shapes were positioned on walls near escape holes. During the test, a bright light with an intensity of 500 lux was illuminated.

The training tests were conducted for 180 s and repeated four times per day (on days 1 to 4). If the mouse was unable to locate the target hole within 180 s, it was guided to the hole by the researcher. On the fifth day, the target hole was closed, and the number of times the mouse crossed the original target hole within 90 s was recorded [56].

#### 4.2.2. Novel Object Recognition (NOR)

The entire task comprised two adaption sessions, which took place on days 1 to 2, followed by a learning phase and a testing phase on day 3. The testing arena used was a plastic box with opaque walls measuring 50 cm × 50 cm × 39 cm. During the adaption sessions, the mouse was placed individually in the center of the arena for a period of 10 min to acclimate. In the learning phase, two identical circular plastic objects were placed on both sides of the floor. The mouse was allowed to freely explore the test apparatus for 5 min. This was followed by the testing phase, in which a novel object with a wooden surface was introduced to replace the original object on the right side for 5 min. Both objects were cleaned with diluted ethanol to eliminate any olfactory cues.

### 4.3. Data Acquisition and Analysis

#### 4.3.1. DEGs

Sequencing analysis for the three groups used ballgown and DESeq2 package R programming software version 1.40.2 to estimate the expression levels of all transcripts and perform expression levels for mRNAs by calculating FPKM (Fragments Per Kilobase of exon model per Million mapped reads). The DEGs were selected with a *p*-value < 0.05 and an absolute value of log_2_ fold change (FC) > 1 were deemed statistically significant in the differential analysis. Specifically, we set the threshold conditions such that a log_2_FC > 1 indicated upregulated genes and a log_2_FC < −1 indicated downregulated genes.

#### 4.3.2. Venn Diagram

The co-expression of genes for each group in the hippocampus and PFC was identified by generating a Venn diagram using Omic Studio https://www.omicstudio.cn (accessed on 19 April 2023), an online tool for multivariate data analysis and visualization.

#### 4.3.3. Heatmaps

Significant DEGs related to the pathways were visualized using interactive heatmaps. Both visualizations were created using the online platform of Omic Studio. Heatmaps were generated by uploading the FPKM values for the three groups on the website.

#### 4.3.4. GO and KEGG Analysis

Functional enrichment analysis, including GO functional annotation and KEGG pathway enrichment analysis, was conducted for DEGs. GO annotation encompassed the analyses of BP, CC, and MF. KEGG analysis was used to identify significantly enriched genetic signaling pathways. This analysis was performed using the “Omic Studio” web platform. The significant enrichment threshold was set at *p* < 0.05, and the top 10 functions and top 20 pathways with the lowest *p*-values are displayed.

### 4.4. Real-Time Quantitative Polymerase Chain Reaction (RT-qPCR) Assay

Total mRNA was extracted from the mouse brain tissue using a kit for rapid mRNA extraction, and cDNA was synthesized following the manufacturer’s instructions and guidelines using the Reverse Transcription System Kit (Yeasen Biotechnology, Shanghai, China). Reactions were conducted using SYBR^®^ Green PCR Master Mix in a QuantStudio 6 Flex sequence detection system (Thermo Fisher, Waltham, MA, USA). Oligonucleotides were designed and synthesized by Sichuan Shenggong Technology Co., Ltd. (Chengdu, China). The samples were analyzed in duplicate and normalized to GAPDH. The expression level of each target gene was analyzed using the 2^−ΔΔCt^ method. The primer sequences used are listed in Table 1.

### 4.5. Statistical Analysis

Data were analyzed using a one-way analysis of variance with Tukey’s multiple-comparison test, and the data are expressed as the mean ± standard deviation. For time-dependent analysis, two-way analysis of variance (ANOVA) was employed in conjunction with Tukey’s post-hoc comparison. Statistical significance was defined as a *p*-value < 0.05. All statistical analyses were performed using GraphPad Prism, version 8.0. Differences were considered statistically significant at *p* < 0.05. Significance levels are denoted as follows: * *p* < 0.05, ** *p* < 0.01, and *** *p* < 0.001.

## 5. Conclusions

In conclusion, our research, based on RNA-seq analysis and RT-qPCR, validated significantly dysregulated rescued DEGs that play a pivotal role in neuroinflammation and the NLR signaling pathway. This provides substantial evidence regarding the intricate processes involved in aging and related cognitive declines. Ultimately, it enhances our understanding of exercise training as a potent and effective intervention for extending both lifespan and health span during aging.

## Figures and Tables

**Table 1:** The primer sequences in RT-qPCR.

Gene	Primer (5′ to 3′)
*caspase1*	F: ACAAGGCACGGGACCTATG R: TCCCAGTCAGTCCTGGAAATG
*Gbp3*	F: TTCACCAACGGCAAGACCAAGACTC R: GTAGCCCAGCTCAATCTTCTTCCTG
*Gbp5*	F: CAGACCTATTTGAACGCCAAAGA R: TGCCTTGATTCTATCAGCCTCT
*Ifi27l2a*	F: GCTTGTTGGGAACCCTGTTTG R: GGATGGCATTTGTTGATGTGGAG
*Ifit1*	F: TTCCGTAGGAAACATCGCGT R: CATGAATGGCCTGTTGTGCC
*Ifi204*	F: CCAGTCACCAATACTCCACAG R: GAGCACCATCACTGTCAGG
*Irf7*	F: GAGCGAAGAGAGCGAAGAGG R: GGCCCACAGTAGATCCAAGC
*Nek7*	F: GCTGTCTGCTATATGAGATGGC R: CCGAATAGTGATCTGACGGGAG
*Nlrp6*	F: CTCGCTTGCTAGTGACTACAC R: AGTGCAAACAGCGTCTCGTT
*Oas1b*	F: GGCCTCTAAGGGGGTCAAG R: CTGGCAGCACGTCAAACTTC
*Septin2*	F: GCCCAGCAACAAGCAAAGCACAT R: CCCAACCACCCTAGTTCCTCCG
*GAPDH*	F: GAACGGGAAGCTCACTGG R: GCCTGCTTCACCACCTTCT

## Data Availability

The data underlying this article are available in the article and in its online supplementary material.

## Supplementary Materials

The following supporting information can be downloaded at: https://www.mdpi.com/article/10.3390/life13101988/s1, Figure S1: GO enrichment in NC vs. NA and NA vs. NE and in the PFC; Figure S2: GO enrichment in NC vs. NA and NA vs. NE and in the Hippocampus; Table S1: Sixty target genes of exercise were associated with aging in the PFC; Table S2: 65 overlapping genes related to exercise and aging in the hippocampus; Table S3: GO and KEGG information in the PFC; Table S4: GO and KEGG information in the hippocampus.
