# Supplementary figures and images for: Bioinformatics Analysis and Experimental Verification of Exercise for Aging Mice in Different Brain Regions Based on Transcriptome Sequencing
Source: Life (Basel). 2023 Sep 29;13(10):1988. doi: 10.3390/life13101988 (PMC10608440; doi:10.3390/life13101988)

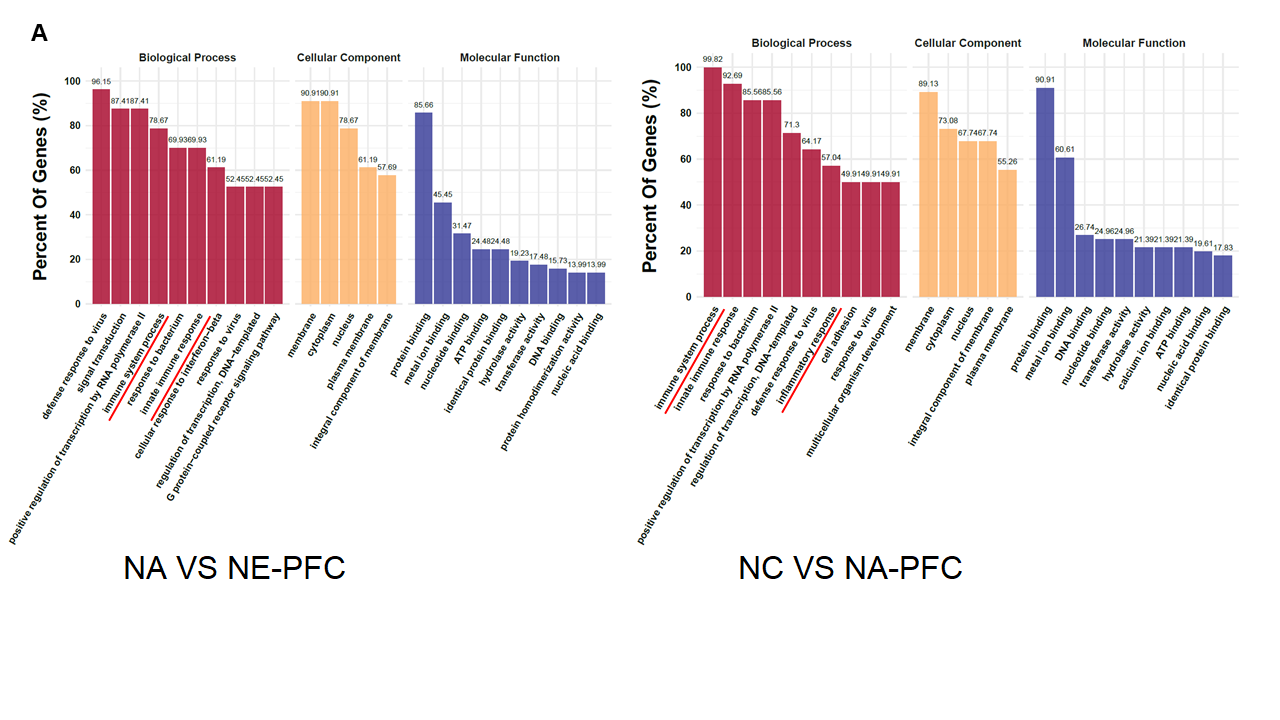

Supplement: Supplementary file 1 [file life-13-01988-s001.zip › Figure S1 GO-PFC.tif]

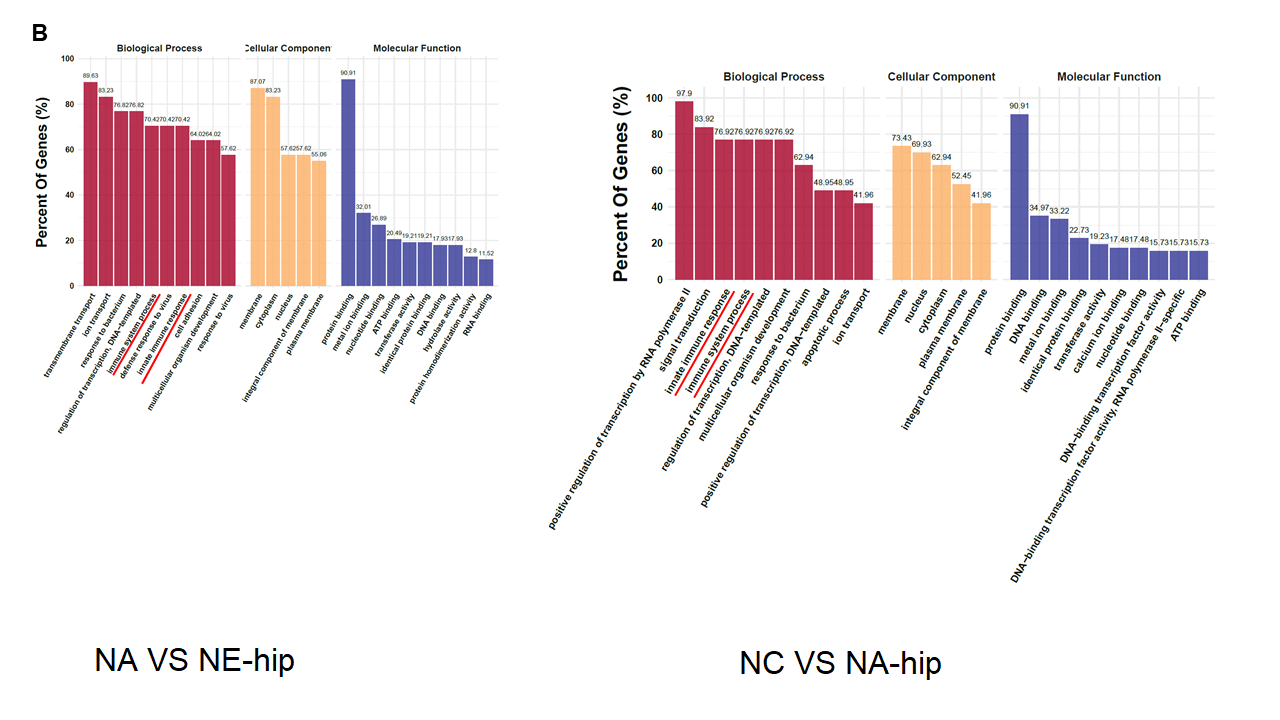

Supplement: Supplementary file 1 [file life-13-01988-s001.zip › Figure S2 GO-hip.tif]
